# Supplementary material for: Characterization and Expression Analysis of Mollusk-like Growth Factor: A Secreted Protein Involved in Pacific Abalone Embryonic and Larval Development
Source: Biology (Basel). 2022 Oct 1;11(10):1445. doi: 10.3390/biology11101445 (PMC9598359; doi:10.3390/biology11101445)
Supplement: Supplementary file 1 [file biology-11-01445-s001.zip › biology-1916340-supplementary.pdf]

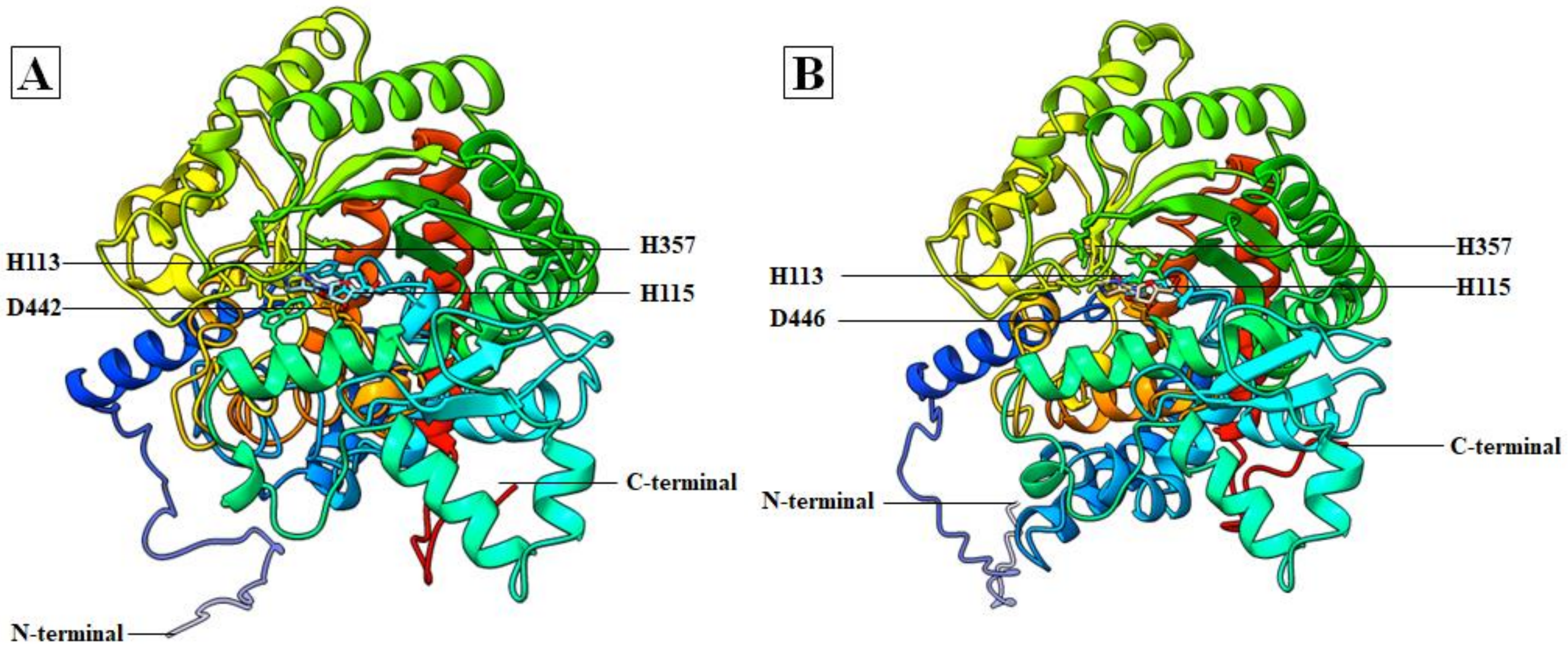

Figure S1. Three-dimensional structure of Pacific abalone (**A**) Hdh-MLGF and disc abalone (**B**) Hdd-MLGF

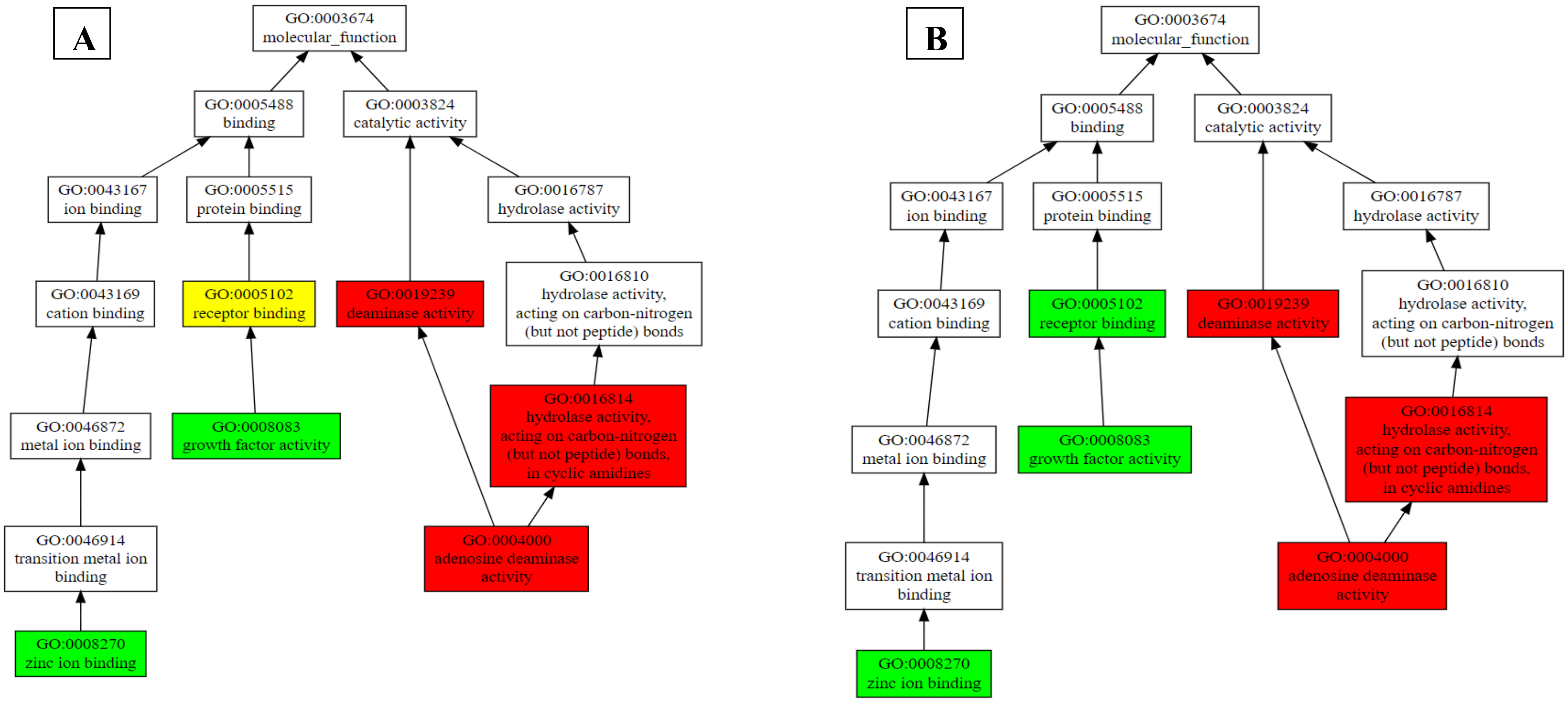

Figure S2. Gene ontology (molecular function) of pacific abalone(**A**) Hdh-MLGF *Aplysia* (**B**) Ac-MDGF
